# Being Open to a Process of Learning: The Meaning of Joint Activities With Patients as Narrated by Nursing Staff in Psychiatric Inpatient Care

**DOI:** 10.1111/inm.13431

**Published:** 2024-09-20

**Authors:** Andreas Glantz, Lena Wiklund Gustin, Ingeborg Nilsson, Anna Westerlund, Jenny Molin

**Affiliations:** ^1^ Department of Nursing Umeå University Umeå Sweden; ^2^ Mälardalen University Västerås Sweden; ^3^ UiT/The Arctic University of Norway Narvik Norway; ^4^ Department of Community Medicine and Rehabilitation Umeå University Umeå Sweden; ^5^ Department of Epidemiology and Global Health Umeå University Umeå Sweden; ^6^ Department of Clinical Science Psychiatry Umeå University Umeå Sweden

**Keywords:** hermeneutics, nurse–patient relations, psychiatric nursing, psychosocial care, social participation

## Abstract

Forming therapeutic relationships is considered important within psychiatric and mental health nursing. One way of achieving this is through social interaction when engaging in joint activities. However, introducing and using nursing interventions based on joint activities in psychiatric inpatient care has proven challenging. Since staff motivation is important, researching the meaning of engaging in joint activities from the nursing professional's point of view can provide information that is relevant in this area. This study aims to illuminate the meaning of participating in joint activities with patients as narrated by nursing professionals in psychiatric inpatient care. Narrative interviews with 14 nursing professionals with experience from psychiatric inpatient care were conducted. Data were analysed using phenomenological hermeneutics and reported following the consolidated criteria for reporting qualitative research (COREQ) guidelines. Results illuminate that engaging in joint activities means being open to a process of learning. Four themes contributed to this understanding: Struggling with uncertainty, discovering aspects of the other, unfolding paths to self‐fulfilment and sharing personhood. Being open to a process of learning means being willing to face uncertainty when engaging in activities as well as being open to learning about oneself as well as the other. Through openness to this process of learning, a sharing of personhood can be achieved, where the nursing professional and the patient approach becoming two persons. Illuminating the meaning of joint activities from the nursing professionals' perspective may provide valuable insights related to introducing and using interventions focusing on joint activities.

## 1 Introduction

In health care, the term ‘activities in everyday life’ is often associated with basic self‐care tasks such as dressing, showering or personal hygiene. However, a crucial but sometimes overlooked aspect is social interaction. For individuals undergoing psychiatric inpatient care, interacting with staff is central, as patients view them as a means to establish mutual trust and a sense of safety. Thus, social interaction is not just something that keeps patients occupied but is an important part of developing therapeutic relationships. Conversely, a lack of interaction may lead to feelings of invisibility and self‐reliance (Molin, Graneheim, Lindgren 2016; Molin et al. 2016).

One effective approach to fostering social interaction is through joint activities. Various nursing interventions specifically target social interactions (Kent 2004; Molin et al. 2018), however introducing and using such interventions can be challenging. Nursing professionals' commitment to engage in joint activities is a critical factor influencing the introduction and use of these interventions. There is a lack of studies illuminating the meaning of nursing professionals participating in joint activities with patients in psychiatric inpatient care. This study aims to contribute to bridging that gap.

## 2 Background

In recent years, voices within psychiatric care have advocated for a shift away from an overreliance on medication, emphasising a move towards person‐centred care that prioritises the development of therapeutic relationships (Bacha, Hanley, and Winter 2020; Gabrielsson et al. 2020; Wand, Glover, and Paul 2022). Patients expect the nursing professionals to facilitate interactions, emphasising the importance of care grounded in humanistic values (Moreno‐Poyato et al. 2016). Even during one‐on‐one observations, interactions and conversations centred around joint activities prove beneficial, fostering therapeutic relationships (Insua‐Summerhays et al. 2018).

Patients often perceive everyday life in psychiatric inpatient care as characterised by passivity, waiting and unclear structures (Lindgren, Aminoff, and Graneheim 2015). Nursing professionals share this perspective, noting that everyday life lacks clear guidance regarding the content of care and requiring them to manage chaos within the wards (Molin, Graneheim, Lindgren 2016; Molin et al. 2016).

Interacting with patients in inpatient psychiatric care is consistently emphasised as crucial by nursing professionals (Gabrielsson, Sävenstedt, and Olsson 2016; Graneheim et al. 2014; Salzmann‐Erikson, Rydlo, and Wiklund Gustin 2016). Current research often emphasises specific activities when investigating the nature of interaction, such as, for example, creative activities, rather than delving into the broader aspects of engaging in joint activities, regardless of the specific activity content involved (Hansen, Erlandsson, and Leufstadius 2021). While there are interventions that enhance opportunities for patient‐staff engagement and thus foster social interaction, the care culture and staff attitude towards activities in psychiatric inpatient care influence whether these can be successfully introduced (Dodd et al. 2018; Salberg et al. 2018).

However, introducing and using these interventions has proven challenging and is dependent on various factors, including the commitment of the staff (Dodd et al. 2018), the staff's attitude towards change as well as towards the intervention itself (Salberg et al. 2018). Furthermore, values and beliefs of the individuals involved in using interventions are central to the willingness to adopt new innovations (Björkdahl et al. 2023). Staff also seem conflicted regarding their view on these interventions. While some view the interventions as ways to foster relationships, others view some activities as having less value and contributing to the ward becoming too cosy and patients not wanting to be discharged (Molin et al. 2020).

In nursing science literature, the care culture has been examined from several points of view. From a theoretical standpoint, having time for the patients and creating therapeutic relationships while providing honest engagement is important (Wangel et al. 2024). Nevertheless, patients report that while this is important also from their perspective, it is not always something that is offered within psychiatric inpatient care. Patients often report feeling isolated from nursing professionals, experiencing a minimum of interactions and a lack of collaboration. They also perceive the nursing professionals as paternalistic and intimidating (Moreno‐Poyato et al. 2016). Although nursing professionals describe themselves as having the patients' best interests at heart, there still seems at times to be a discrepancy between what nurses describe and patients perceive (Molin, Graneheim, Lindgren 2016; Molin et al. 2016).

There is a lack of knowledge regarding the meaning of nursing professionals participating in joint activities with patients. Since staff motivation and commitment are important for the care culture, illuminating the meaning of joint activities from the nursing professionals' perspective may provide valuable insights.

### 2.1 Aim

The aim of this study was to illuminate the meaning of participating in joint activities with patients as narrated by nursing professionals in psychiatric inpatient care.

## 3 Methods

This qualitative study utilised narrative interviews with nursing professionals and data were interpreted using phenomenological hermeneutics.

### 3.1 Setting

The interviews focused on the meaning of nursing professionals engaging in joint activities within psychiatric inpatient care in Sweden. These wards often specialise in the treatment of various disorders, including affective, psychosis or substance use disorders. The participants in this study had experience working in a variety of wards with different specialisations.

### 3.2 Participants

Fourteen nursing professionals participated in this study. The inclusion criterion was experience from working in adult psychiatric inpatient care as either registered nurses or enrolled nurses, irrespective of past or recent work experience, as well as a willingness to share experiences of joint activities. Participants were recruited by advertising in social media aimed at psychiatric and mental health nursing in Sweden. Some of the participants contacted the researchers directly, while others signed up for more information through a website provided by the research team. The participants came from different parts of Sweden and were between 33 and 76 years of age (median 43.5 years). Nine were female and five were male and they had experience of working in psychiatric inpatient care ranging from 1 to 33 years (median 11 years). Nine were registered nurses and five were enrolled nurses. Seven of the registered nurses had advanced level education in psychiatric and mental health nursing.

### 3.3 Data Collection

Narrative interviews (Mishler 1986) were conducted by the first author, who is a male registered nurse with a master's degree in nursing science and advanced level education in psychiatric and mental health nursing as well as psychotherapy. He is currently a doctoral student and has training in interviewing for qualitative studies. The interviews were conducted from March through April 2023. One of the interviews was carried out face to face, while all other interviews took place online. The participants had prior to the interviews filled out a form with demographic questions. The interviews began with an open question—‘Can you tell me about a situation where you engaged in a joint activity together with a patient?’. Follow‐up questions such as ‘What made you think/feel/do that?’ and ‘What happened next?’ were posed to support narration.

The definition and interpretation of ‘joint activity’ were left open to the participants. The interviews addressed a range of situations, spanning from past occurrences to more recent events and included activities such as walks, conversations and playing games or engaging in creative activities. The interviews lasted from approximately 21 to 59 min (median 34.5 min) and were recorded digitally and transcribed verbatim by the first author.

### 3.4 Interpretation

The interpretation of the narrative interviews was approached through phenomenological hermeneutics inspired by Lindseth and Norberg (2004, 2022). The interpretation was a process moving in a hermeneutic arc between understanding, explanation and new understanding (Ricœur 1991). Initially, all the transcripts were read through several times and a naïve understanding of the text was formulated. This naïve understanding represented an initial idea of the meaning of nursing professionals engaging in joint activities with patients in psychiatric inpatient care. Then, a structural analysis of the transcripts followed where the text was taken out of context and viewed as standing on its own. This decontextualisation aims at creating a distance to the researcher's preunderstanding by explaining the structure of the text (Ricœur 1991). This is accomplished by identifying the themes around which the narratives are constructed. The condensed meaning units were organised and through further abstraction, eight subthemes and four themes were developed. By reflecting upon the result of the analysis, through the relationship between naïve understanding, structural analysis and theory, results were recontextualised and a new, comprehensive understanding emerged, making the result understandable in a wider context. The first author carried out the analysis, while continuously discussing the interpretation with the other authors. The study adhered to the consolidated criteria for reporting qualitative research (COREQ) (Tong, Sainsbury, and Craig 2007).

### 3.5 Ethical Considerations

While there is a potential for participants to feel assessed during the description of work‐related activities, Gaydos (2005) notes that personal narratives foster meaningful connections between narrators and listeners, offering both relief and gratitude. Confidentiality adhered to current regulations, with interview statements potentially identifying participants (e.g., names of places or hospitals) excluded from transcriptions. Demographic details are only provided at a general level to prevent identification. Participants from various regions of Sweden further mitigated the risk of identification. Participants received written and verbal study information, and those who opted to participate provided written informed consent. This study was approved by the Swedish Ethical Review Agency (Dnr. 2022‐06774‐01).

## 4 Results

The findings of this study are presented as a naïve understanding, the structural analysis and a comprehensive understanding, inspired by the process outlined by Lindseth and Norberg (2004, 2022).

### 4.1 Naïve Understanding

The meaning of joint activities encompasses curiosity that stems from the opportunity to learn something new about another person while also experiencing joy and having fun together. It reveals a different side of the other, leading to a sense of unity and togetherness. This can mean genuineness and the transition from being merely staff members to being individuals. At the same time, it can also mean seeing the healthy amidst the illness. This occurs through engaging in activities as a way to focus on and be attentive to the healthy aspects. It involves setting aside illness, medication and diagnoses, and engaging in things related to everyday life. This means being spontaneous and working with the available resources. It can also mean showing courage when taking risks based on the patient's health condition and having to justify one's decisions to the rest of the staff. Additionally, joint activities also mean having opportunities for exploring daily living capabilities and helping nursing professionals gain clarity both professionally and personally. When an activity is perceived as successful, it can also evoke a sense of pride.

### 4.2 Structural Analysis

The structural analysis resulted in four different themes, along with subthemes, which are presented in Table 1 and elaborated in the following paragraphs.

**TABLE 1:** Themes and subthemes.

Subtheme	Theme
Daring to take a chance	Struggling with uncertainty
Persevering despite backlash from colleagues	
Opening up for new facets	Discovering aspects of the other
Feeling deceitful in engagement	
Feeling hope and pride	Unfolding paths to self‐fulfilment
Developing a renewed understanding of oneself	
Revealing vulnerabilities	Sharing personhood
Becoming two persons through togetherness	

#### 4.2.1 Struggling With Uncertainty

This theme consists of two subthemes illustrating the uncertainty that nursing professionals face when they engage in joint activities together with patients. This uncertainty means not knowing how a situation will unfold, whether a patient is ready for a certain activity or feeling uncertain whether engaging in a joint activity will result in backlash and criticism from colleagues. Persevering despite this possible backlash means struggling with breaking existing patterns in the culture of care.

##### 4.2.1.1 Daring to Take a Chance

Engaging in joint activities means taking a chance and requires a certain amount of courage. Even though engaging in a joint activity might mean encountering unforeseen situations and dealing with uncertainty, the potential benefit makes taking the chance worthwhile.

The first thought was that I hope I don't bring something up that stirs him up again. But afterwards the patient said that it felt good to talk about it because the music evoked a memory which he had a need to talk to me about. And after the conversation he said that he felt really good talking. That he had repressed some things and the music brought up so many memories of loved ones. […] So that felt good. It felt good for me, and it felt, I think it felt good for the patient too. (Participant 8)

Daring to take a chance also means that, even though there might be a lack of trust and uncertainty about what might happen, an activity such as taking a walk together, will help build trust and outweighs the risks. It also signals that the nursing professional has faith in the patient.

Even though nursing professionals struggle with uncertainty, and the joint activities do not always turn out the way that is anticipated, this does not equal being dissuaded from trying again. It means that the nursing professionals think outside the box and learn something from the situation. Experiencing one unforeseen situation does not mean that it will happen every time and without trying there is no way to know what will happen and what can be learned.

##### 4.2.1.2 Persevering Despite Backlash From Colleagues

Persevering despite backlash means both having a fear of being criticised and experiencing negative comments from other members of the staff when engaging in joint activities with patients. This could be for several reasons, both because they would be unavailable for other work at the ward while the activity was carried out, but also because of others' opinions of the activity or how others perceived the value of the activity.

But I had to defend my actions with the colleagues. […] Because while I was out walking, I couldn't help with the telephones or be available in other ways. […] I don't know if defend is the right word. At least I had to explain myself to a few of them. (Participant 3)

While the need for relationship building and the creation of alliances is undisputed, one criticism from colleagues is that the burden of work weighs heavier on those who do not engage in an activity. This means that persevering despite backlash is about coping with feelings of stress while engaging with a patient in a joint activity.

Persevering despite backlash can also mean that others do not always consider joint activities as ‘proper work’, and that spending time doing activities together is not work that is considered of value. This can in turn lead to conflicts or struggles in the team. Activities such as going on excursions or trips or celebrating midsummer with the patients are not always appreciated by all the members of the staff.

Yet another aspect of this is a concern that if an activity does not work out as planned, this means that others have to get involved which might mean more work. This could mean fewer opportunities for engaging in activities with the patients in the future. Despite the uncertainty of receiving backlash and what that might mean, taking a chance and engaging in activities is still considered worthwhile.

#### 4.2.2 Discovering Aspects of the Other

The next theme describes how the nursing professionals, through joint activities, gain new information about the other person. This can both uncover new aspects and help with assessing the well‐being of the other person but can also be seen as deceitful, as discovering this new information about a patient may not always be perceived as completely honest.

##### 4.2.2.1 Opening Up for New Facets

Joining the patients in activities can reveal something previously not seen and hence open up for new facets, for example, while creating a journal of positive experiences together with the patient.

I got to know her in a completely different way. […] We had many good conversations […] [and] approached subjects that I don't think we otherwise would have approached. (Participant 7)

Getting to know the other person in a different way also means seeing the person behind the illness and exploring their person rather than just the symptoms. Joint activities are experienced as a way to help the other person to open up and facilitate conversations.

Opening up for new facets can also be about seeing how a patient copes with everyday situations. Playing a game together can for instance mean an opportunity for the nursing professionals to see whether the patient can understand the rules. Engaging in a group activity can mean that the nursing professionals experience how well the patient can function in a social context and get a different understanding of the patients' actions, challenges and strengths. While the primary purpose of joining a patient in an activity may not be for assessment, it is perceived as a positive side effect of doing the activity. In this way, joint activities with patients meant taking advantage of the situation and learning something new about the patient.

##### 4.2.2.2 Feeling Deceitful in Engagement

Discovering aspects of the other can mean feeling deceitful when engaging the patient. While nursing professionals believe that patients probably understand that assessments of the patients' health happen, even if that is not the intent of engaging in an activity, there is a feeling that there is something not entirely honest about assessing the patient's actions, challenges and strengths while for instance playing cards.

Some patients want to hide how bad they feel, they don't want to make a thing out of it. And then, if we through a social setting can draw it out of them in a way that they are not aware of, that's not the nicest thing. (Participant 3)

Approaching a patient in private and asking if they want to talk means providing a choice whether to interact or not. But in a social setting that choice is not as obvious or might not be as easy to make. This shows how engaging in a joint activity and discovering new aspects can mean also feeling deceitful towards the other.

#### 4.2.3 Unfolding Paths to Self‐Fulfilment

Unfolding paths to self‐fulfilment is about how engaging in joint activities with patients can mean positive experiences both on a personal and professional level. This theme shows how engaging in joint activities could mean feelings of joy, hope and pride and contribute to a better understanding of oneself.

##### 4.2.3.1 Feeling Hope and Pride

Engaging in joint activities means experiencing happiness and being joyful. This can range from feeling happy when hearing laughter at the ward during activities as well as experiencing having a good time together with the patient. This means that even if an activity could be perceived as simplistic, such as playing cards, it brings something different to the caring rather than just observing and monitoring the patient. Seeing a patient succeed in an activity also brings a sense of hope that is needed when working with patients suffering from severe mental ill‐health.

…It brought a new spark, sort of, that oh, now we've really moved on. […] So for me personally, it kept my hope up, that it would be ok. (Participant 12)

While working in psychiatric inpatient care can be challenging, being able to engage in joint activities brings new energy and shows that the care provided can mean something positive and not just consist of coercion. Experiencing joy and hope while engaging in joint activities also includes experiencing a sense of pride, that caring for the patients in this way has a significant impact and requires professional skill.

No but it felt like this is what my job is all about. […] That in some way, here is where I'm useful. […] I don't think just anyone can do this. I don't know, I think I was a little proud, that we had come this far. (Participant 4)

Leaving the workplace after being able to engage in activities meant a feeling of fulfilment. A feeling of being able to bring something meaningful to the care provided that day enhances the experience of pride and satisfaction of the work and that the care does not only consist of keeping the patients at the ward.

##### 4.2.3.2 Developing a Renewed Understanding of Oneself

This subtheme means that joining in activities teaches the nursing professionals something new about themselves. This learning can be related to both personal and professional development.

So I think I became clearer as both person and professional. That even if I'm a nurse, I can also be unhappily in love, or can't afford to go to a concert or whatever it may be. (Participant 2)

This means a developing and renewed understanding for the similarities that are shared between humans no matter what role you have in a caring situation, but also highlights the differences that exist between a nursing professional and a patient. Finding the commonalities with patients through an activity means creating a unity. Similarly, if a mistake is made or a situation misjudged, with an open enough attitude there is something to be learnt from that as well.

Understanding oneself can also mean learning new things together with the patients, such as learning to cook but also learning to trust in oneself. When doing things that others might be apprehensive of, such as taking walks with a patient they felt uncertain about, self‐confidence increased and led to feelings of safety.

#### 4.2.4 Sharing Personhood

The final theme reveals how engaging in joint activities creates an opportunity for interpersonal understanding and sharing of personhood. Being together and revealing vulnerabilities, the nursing professional shares personhood with the patients that emphasises the reduction of power imbalances and promotes equality.

##### 4.2.4.1 Revealing Vulnerabilities

Joining in activities with patients means being open to revealing one's own vulnerabilities, feeling that the inherent power structures of inpatient psychiatric care can be somewhat challenged. This happens when they feel that they show themselves to be human, with flaws and imperfections just like anyone else.

I got something out of it when they saw me sweating and saw that I definitely couldn't do some of the push‐ups that others could do. We were on the same level… (Participant 14)

Revealing vulnerabilities can also mean doing things that might not be considered healthy, but which show a human or imperfect side of the nursing professional. Even though for instance the negative health aspects of cigarette smoking are undisputed, there is a duality in the act of smoking. Showing a ‘weakness’ together with a patient, such as smoking, also means that the power balance is affected. This can in turn lead to the nursing professional and the patient approaching a state of shared personhood rather than being just the nursing professional and the patient.

##### 4.2.4.2 Becoming Two Persons Through Togetherness

Joining in an activity means that coming together could affect the inherent inequality of inpatient care. The everyday nature of the activity means that focus is less on medicine or illness and that there is an ordinariness in the activity that brings something of an equalisation to the power balance.

Because I mean, then we're sitting there having coffee like two ordinary persons in some way. Even though I'm still in a position of power. But one can do as much as possible to not make it visible towards the patient. (Participant 11)

Engaging in a joint activity means that nursing professionals and patients become just two persons doing what is an ordinary part of everyday life, in contrast to being a professional and a patient. This also promotes the idea of sharing personhood where being a person is central rather than being a role. Minimising the difference between nursing professional and patient and, from an outsider's perspective, not being able to tell the two apart, can be achieved through a simple activity such as taking a walk together outside.

### 4.3 Comprehensive Understanding

When the naïve understanding and the structural analysis were reflected on in the light of each other and theory, a main theme expressed as ‘Being open to a process of learning’ evolved. This learning process is one of increasing knowledge of the other and the self and self‐fulfilment on the path to affecting the relationship from a staff‐patient focused relationship towards a more person–person focused relationship. Being open to this process means being courageous, curious and willing to learn about both oneself and the other.

This process of learning reflects Eriksson's (1987) basic assumption that learning together with playing and tending constitute the caring substance. The joint activities in which nursing professionals engage with patients can be viewed as instances of play. There are according to Eriksson (1987) five basic forms of play where one of them, play as testing and training, is of particular interest in this context. Play as testing and training means an opportunity to practice skills and change, modify and hone different activities. While Eriksson discusses playing from the patient's point of view, there are similarities also when considering the nursing professional's perspective. Being able to play requires trust and can be discerned in the first theme, ‘Struggling with uncertainty’. The nursing professional initially struggles with the uncertainty implicit in taking risks when engaging in joint activities with patients. Taking risks means not knowing how engaging in an activity will turn out but deciding that it is worth taking the risk in order to build trust. Just like how play also means testing and training for patients, it is also an opportunity for nursing professionals to try activities and learn about both themselves and the patients.

Learning as one element of caring means constant change and development that requires trust (Eriksson 1987). It is a process between oneself and others (Bergbom, Nåden, and Nyström 2022), meaning that the learning that the nursing professionals experience when engaging in joint activities might also be a learning opportunity for the patients and increase the togetherness that the professionals experience. The learning of aspects of the other and of the self, as shown in the themes ‘Discovering aspects of the other’ and ‘Paths to self‐fulfilment’ illustrates the importance of the relationship with others. Eriksson (1987) describes the relationship between the self (the patient) and the professional other (the nursing professional) as important when the patient is unable to care for him‐ or herself, or when the care provided by natural others (friends and family) is not enough. We would argue that this dependency on others goes both ways: the self of the nursing professional depends on the caring relationship with the patient to nurture and grow both as persons and as professionals.

The learning process can also be challenging, as the nursing professional's personal development also includes reflections on themselves as deceitful when assessing patients during joint activities. Whether this also leads to nursing professionals choosing to not engage in activities remains unclear. However, such ethical reflections, made by the nursing professional, require a certain degree of openness to learning. This is important in connection to the care culture of psychiatric inpatient care. Nursing professionals describe how ethical reflection is mostly individual and something that happens throughout the day (Molewijk, Hem, and Pedersen 2015). While the possible element of deceit in engaging in joint activities and hence making assessments might be considered a transparency issue, it is important that ethical reflection is possible and encouraged in psychiatric inpatient care units. This can aid the nursing professionals in being aware of what their actions might mean and prevent a progression towards situations where joint activities are used in a deceitful way, as this could negatively affect the power balance and make it more difficult to achieve mutual trust.

The meaning of the nursing professionals engaging in joint activities with patients can be seen as culminating in the sharing of personhood between the nursing professionals and the patients. Caring is, according to Eriksson, sharing (Bergbom, Nåden, and Nyström 2022). This includes the nursing professionals being able to reveal their own vulnerabilities. Revealing vulnerabilities can mean that they show themselves as genuine and being wholly themselves, something that Rogers (1957) highlights as one of the necessary conditions for therapeutic change. Eriksson also underlines the importance of authenticity or genuineness when describing ‘natural caring’ (Bergbom, Nåden, and Nyström 2022). Natural care is, according to Eriksson (1987), the core or purpose of professional care, and an important part of natural care is friendship. Friendship is defined by her as standing next to each other in a living meeting. Caring as tending, playing and learning emerges between the self and the other and mutuality is a uniting factor in the natural care. Eriksson (1987) writes that friendship in a professional context is possible and can be beneficial. It would seem that being open to learning about oneself and about the other is an important prerequisite to the sharing of personhood and approaching what could be seen as professional friendship.

## 5 Discussion and Methodological Considerations

The comprehensive understanding, which constitutes a summarising and in‐depth understanding of the themes of the structural analysis, showed that being open to a process of learning requires both a willingness in the individual as well as a caring culture in the organisation where this openness can be allowed to thrive and develop. This makes it essential to finally reflect on learning in relation to the caring culture because nurses gain knowledge of the care culture at their workplace through listening, observing and also adapting to the current culture, a culture which can be either beneficial or detrimental to good care (Rytterström, Cedersund, and Arman 2009). This is in line with Eriksson's description of a caring culture as shaped by humans and either more or less healing (Bergbom, Nyström, and Nåden 2022).

If we focus on joint learning as part of a caring culture, we can also reflect on the importance of addressing this when striving to bring theory closer to practice. In forensic psychiatric care, tensions between different perspectives have been shown to constitute an obstacle to caring (Hörberg 2018). This makes it reasonable to assume that different views on joint activities can not only create the tensions that appear in the results but also complicate the integration of theory and practice, as it may be more difficult to integrate theory that challenges one's own and the culture's understanding with practice.

The study highlights predominantly positive experiences resulting from engaging in joint activities with patients. Nevertheless, some participants recounted instances where colleagues regarded such activities as trivial or not aligned with ‘proper work’. Notably, narratives portraying joint activities as undesirable or detrimental were absent in this study. While both positive and negative views of joint activities in psychiatric inpatient care were requested during the interviews, it is plausible that nursing professionals with negative views opted not to partake in the study. As seen in the narratives told by participants, having a negative view on joint activities is not entirely uncommon. This limitation suggests that valuable insights into the less positive aspects of engaging in joint activities might not be fully represented in our findings.

Some participants' narratives took place several years in the past. While the passing of time might affect recall and distort the recollection of details of past experiences, the purpose of this study was not to elicit exact statements regarding certain situations, but rather to illuminate the meaning of engaging in joint activities. The importance of the memory of a situation lies not necessarily in the accuracy of the recalled details (Gaydos 2005). The passing of time from when a situation occurred to when it was recalled in an interview may lead to integration, providing an opportunity for reflection and meaning making. This may also lead to the person being interviewed gaining insights into the significance of the situations as well as a deeper understanding regarding the context surrounding these past situations which is advantageous for this kind of study. Situations that have special meaning for an individual are also probably more likely to be remembered.

A text can be interpreted in different ways depending on, for example, the researchers' preunderstanding and other interpretations could be possible (Lindseth and Norberg 2022). Using phenomenological hermeneutics and moving between understanding‐explanation‐new understanding de and recontextualises the text and makes it applicable in a wider context. Transferability is also dependent on the readers’ interpretation of our text, that is, the findings, in relation to their own contexts.

## 6 Conclusion

Engaging with patients in joint activities means being open to a process of learning. This means being open to facing a potential struggle with the uncertainties of engaging in activities, both in terms of not knowing how the engagement will turn out, but also in not knowing whether one will be criticised for spending time in activities with patients or not. The process of learning also leads to discovering new aspects both of the patient and of oneself and can lead to self‐fulfilment and highlights the importance of the relational character of caring. In the end, being open to a process of learning leads to a sharing of personhood where the nursing professional and the patient approach being two persons, joined in a professional friendship.

## 7 Relevance for Clinical Practice

This study shows that engaging in joint activities with patients means being open to a learning process. It shows the importance of joint activities both for the development and learning of the nursing professionals as well as for creating relationships. This is relevant knowledge when introducing and using nursing interventions that focus on social interaction and joint activities since motivation and perceived importance of the intervention are important factors to consider. It also highlights the importance of a care culture that is permissive towards social interaction and activities in order for nursing professionals to be able to develop this openness to learning more about themselves and patients. It might also in the long run support the development of therapeutic relationships.

## Author Contributions

All authors listed meet the authorship criteria according to the guidelines of the International Committee of Medical Journal Editors, and all agree with the submitted manuscript.

## Ethics Statement

This study was approved by the Swedish Ethical Review Authority (Reg. no. 2022‐06774‐01).

## Conflicts of Interest

The authors declare no conflicts of interest.

## Data Availability

The data that support the findings of this study are available on request from the corresponding author. The data are not publicly available due to privacy or ethical restrictions.
